# A comprehensive pan-cancer analysis of the expression characteristics, prognostic value, and immune characteristics of *TOP1MT*

**DOI:** 10.3389/fgene.2022.920897

**Published:** 2022-08-10

**Authors:** Lihong Fei, Zhimin Lu, Yufen Xu, Guoxin Hou

**Affiliations:** ^1^ Department of Gastroenterology, The First Hospital of Jiaxing, Affiliated Hospital of Jiaxing University, Jiaxing, Zhejiang, China; ^2^ Department of Outpatient, The First Hospital of Jiaxing, Affiliated Hospital of Jiaxing University, Jiaxing, Zhejiang, China; ^3^ Department of Oncology, The First Hospital of Jiaxing, Affiliated Hospital of Jiaxing University, Jiaxing, Zhejiang, China

**Keywords:** *TOP1MT*, pan-cancer, prognosis, biomarker, immunity

## Abstract

**Background:** Mitochondria are at the heart of a number of metabolic pathways providing enormous energy for normal cell growth and regulating tumor cell growth as well as survival. Mitochondrial topoisomerase I (*TOP1MT*) is a type IB topoisomerase found in the mitochondria of vertebrates. However, no pan-cancer analysis of *TOP1MT* has been reported. This study aims to explore *TOP1MT* expression in pan-cancer tissues and identify whether it can be a target for mitochondrial anticancer therapy.

**Methods and results:** The original *TOP1MT* expression data in 33 different types of cancer patients were downloaded from the TCGA and GTEx databases. *TOP1MT* was highly expressed in cancer tissues, including BLCA, BRCA, CHOL, COAD, DLBC, ESCA, GBM, HNSC, KIRC, KIRP, LGG, LIHC, LUAD, LUSC, PAAD, PCPG, PRAD, READ, SKCM, STAD, THYM, UCEC, and UCS. According to Kaplan-Meier survival curve analysis, high *TOP1MT* expression in BLCA, HNSC, KIRP, PAAD, UCEC, and LIHC cancer tissues was linked to poor prognosis of cancer patients, i.e., poor OS, disease-specific survival, and PFI. Linkedomics analysis identified a positive correlation of *TOP1MT* expression with CNA, but a negative correlation with methylation. *TOP1MT* expression significantly correlated with immune cells and immune checkpoints in the TIMER database. Functional analysis showed a close relationship between *TOP1MT* expression and ribosomes.

**Conclusion:** In summary, *TOP1MT* is a potential biomarker for mitochondrial anticancer therapy and cancer immunotherapy.

## Introduction

The primary role of mitochondria in the human body is the production of Adenosine Triphosphate (ATP) and the synthesis of metabolites necessary for cell bioenergy and biosynthesis (Chandel Navdeep, 2015). Mitochondria generate ATP and metabolites through glycolysis, amino acid decomposition, and fat decomposition, among other processes. These metabolites enter the mitochondria via the Tricarboxylic acid cycle (TCA cycle), producing Nicotinamide adenine dinucleotide (NADH) and reductive molecules, including Flavine adenine dinucleotide, reduced (FADH-2), NADH Subsequently, these energy molecules are phosphorylated via oxidation to produce ATP, whereas the TCA cycle-generated intermediates can be collected and used in other biosynthetic metabolic pathways to produce glucose, amino acids, nucleotides, and other important substances (Spinelli and Haigis, 2018). In this regard, mitochondria regulate several metabolic pathways, supplying enormous energy to cells, which is important for cell growth, survival, and death. Recent evidence indicates that mitochondria promote tumor cell growth and survival in the pathological microenvironment; they also provide tumor microsynthesis and biological energy requirements. This provides a theoretical reference for targeting mitochondria in anticancer therapy. Also, mitochondria are potential targets for the development of novel anticancer drugs (Vyas et al., 2016; Porporato et al., 2017).

Topoisomerase is a ubiquitous enzyme that instantly splits single or double chains in the phosphodiester framework of nucleic acid, regulating the topological structure of nucleic acid (Wang, 2002). Human topoisomerases are classified into two types, i.e., type I and II (Forterre et al., 2007; Forterre and Gadelle, 2009; Singh et al., 2020). Type I topoisomerase instantly cleaves one strand of double-stranded DNA, allowing the other single strand to pass through the gap, thereby disrupting the situation of a DNA superhelix or helix deletion. II-type topoisomerase uniformly cuts both chains and then connects the broken ends. Type I is divided into Type IA (*TOP3α* and *TOP3β*) and Type IB (*TOP1, TOP1MT*), whereas type II is divided into Type IIA (*TOP2α* and *TOP2β*) (Hou et al., 2017; Zhang et al., 2017). *TOP2α, TOP2β,* and *TOP3α* are located in both nucleus and mitochondria (Low et al., 2003; Zhang et al., 2014; Nicholls et al., 2018). *TOP1MT* is the only topoisomerase located in the mitochondria (Zhang et al., 2001; Pommier et al., 2016). Unlike other eukaryotic organelles, mitochondria have their circular DNA (mtDNA), encoding 13 oxidative phosphorylation complex proteins. One recent study discovered that *TOP1MT* is critical in maintaining the integrity of mtDNA and limiting the negative superhelix of mtRNA (Seol et al., 2012; Zhang et al., 2014). *TOP1MT* maintains tumor cell proliferation and promotes tumor growth in mice models of colon and liver cancers with impaired metabolism (Baechler et al., 2019). *TOP1MT* is unable to inhibit liver cell proliferation in the liver regeneration model by limiting mtDNA copy number amplification (Khiati et al., 2015). Besides the effects listed above, *TOP1MT* modulates mitochondrial functional pathways (Zhang and Pommier, 2008; Dalla Rosa et al., 2017; Baechler et al., 2019). Since tumor cells highly depend on mitochondrial biology and *TOP1MT* could be a target of cancer drugs, inhibiting *TOP1MT* could be an effective approach for eliminating cancer cells (Scatena et al., 2018).

Herein, we analyzed *TOP1MT* expression features, prognostic value, correlation with tumor immune infiltrating cells, and biological function using data obtained from cancer genome map (TGCA), TIMER, linkedomics, cBioPortal, and TISIDB databases. *TOP1MT* regulates tumor development and is a potential biomarker for targeted mitochondrial anticancer therapy.

## Materials and methods

### *TOP1MT* expression pattern in human pan-cancer

Gene expression data from normal tissues in the GTEX database (http://gtexportal.org/) were integrated with gene expression data from more than 11,000 tumors of 33 cancer types in the cancer genome atlas (TCGA https://www.cancer.gov/) to analyze and compare the *TOP1MT* expression pattern in human pan-cancer tissues and normal tissues. Sample size of 33 different tumor types were shown in Supplementary Table S1. Further, we analyzed the correlation of *TOP1MT* expression in TGCA with different pathological stages of tumor cells. The log2 transformation was used to normalize all expression data. *t*-test, *p* < 0.05 was used to determine statistical significance.

### Prognosis

The forest graph was used to examine the relationship between *TOP1MT* expression and the prognosis of 33 different types of cancer patients. The relationship between *TOP1MT* expression and prognosis of patients with various cancer and pathological stages was further investigated using the Kaplan-Meier curve, which included total survival (OS), disease-specific survival (DSS), and progression-free periods (PFI). The risk ratio (HRS) and 95% confidence interval were calculated using a single factor survival analysis.

### Linkedomics

The linkedomics database (http://www.linkedomics.org/) was used to investigate the methylation level and copy number variation data levels of *TOP1MT* in various tumors. Further, the relationship between *TOP1MT* expression, methylation level, and copy number variation of *TOP1MT* was analyzed in tumor tissues. Statistically significant differences were estimated by *t*-test (*p*＜0.05).

### Gene enrichment analysis

Pan-cancer *TOP1MT* co-expressed genes for gene enrichment analysis were selected to understand the biological function and pathway involved in *TOP1MT* through TCGA database. The potential pathways were identified by the gene ontology (GO) terminology and the KEGG pathway enrichment analysis. The three categories of GO analysis included biological processes (BP), cellular components (CC), and molecular functions (MF).

### Immune infiltration

The TIMER database (https://cistrome.shinyapps.io/timer/) was used to analyze the relationship between immune infiltrating, *TOP1MT* expression and through TISIDB (http://cis.hku.hk/TISIDB/) analysis *TOP1MT* gene expression in the different immune subtypes.

### cBioPortal

cBioPortal (https://www.cbioportal.org/) is a repository of cancer genome data. *TOPIMT* copy number alterations (CNA) were investigated in various cancers.

### Statistical analysis

The *t*-test was used to estimate *TOP1MT* expression in cancer and normal tissue. Single Factor Cox regression analysis was used to calculate the HR and P. Kaplan-Meier analysis was used to stratify *TOPIMT* expression. The Spearman correlation analysis was used to evaluate the correlation of *TOP1MT* expression with the level of methylation and the number of gene amplification. All analyses were performed with R software (version 3.6.3). *p* ＜0.05 was used as the significance threshold of statistical analysis.

## Results

### *TOPIMT* expression in human carcinomatous tissues

**FIGURE 1:**
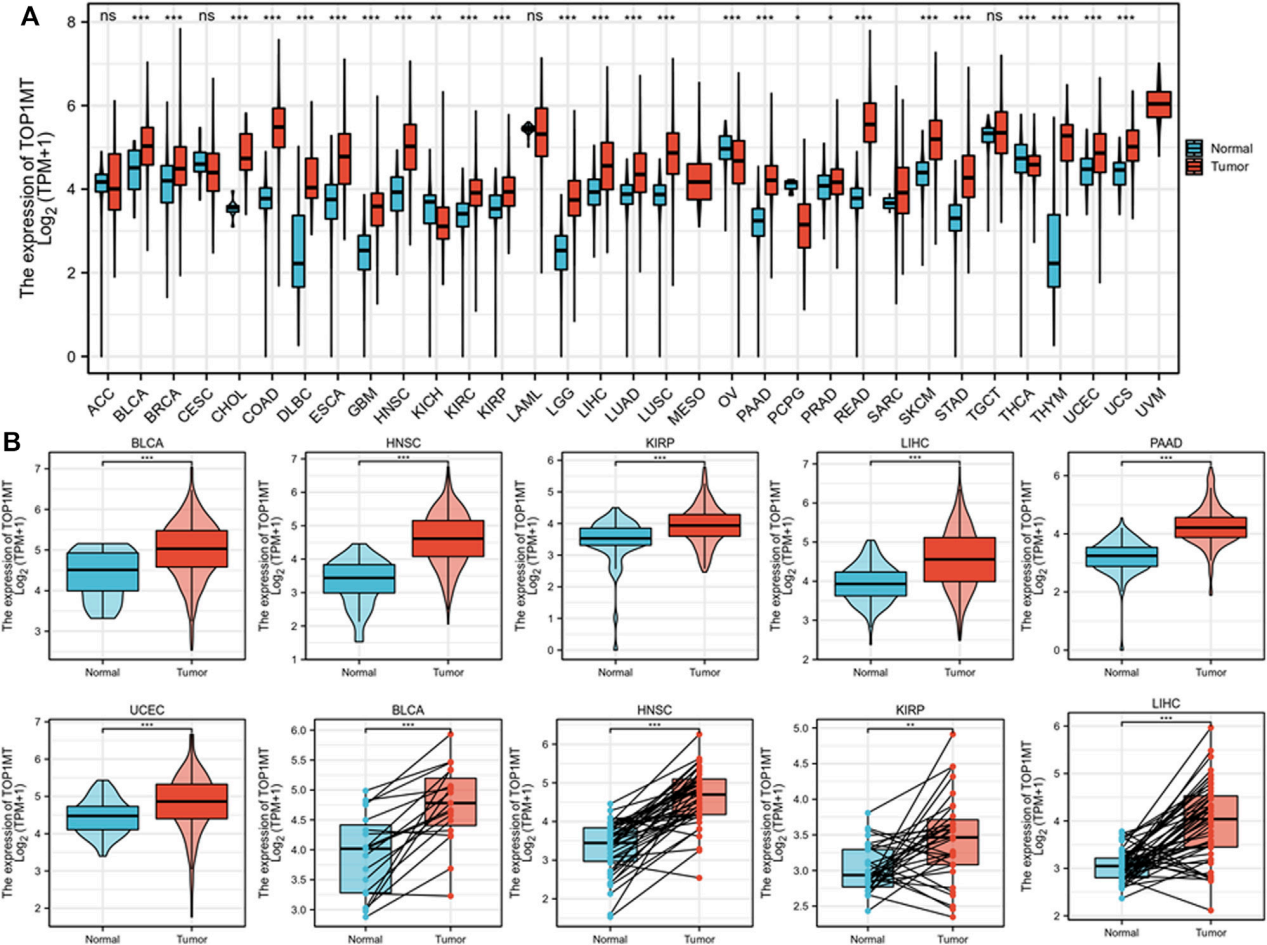
Expression pattern of *TOP1MT* in human generalized carcinoma. **(A)** Assessing mRNA expression of *TOP1MT* between tumor and normal tissue using data downloaded from TCGA and GTEx; **(B)** *TOP1MT* mRNA expression in patients with BLCA, HNSC, KIPP, PAAD, UCEC, and LIHC from TCGA. Log_2_ (TPM+1) is used for the logarithmic scale. ∗ *p* < 0.05, ∗∗ *p* < 0.01 and ∗∗ *p* < 0.001.

To explore the role of *TOPIMT* in cancer, tumor samples from the TGCA were combined with normal samples from GTEx to characterize the *TOPIMT* mRNA expression. As shown in Figure 1A, *TOP1MT* expression was consistently higher ion in tumor samples from BLCA, BRCA, CHOL, COAD, DLBC, ESCA, GBM, HNSC, KIRC, KIRP, LGG, LIHC, LUAD, LUSC, PAAD, PCPG, PRAD, READ, SKCM, STAD, THYM, UCEC, UCS than in normal tissues (Figure 1B, Supplementary Figure S1A). However, no difference was noted in *TOP1MT* expression between ACC, CESC, LAML, and TGCT tumor samples and normal tissues (Supplementary Figure S1B). Lower *TOP1MT* expression was discovered in tumor samples from KICH, THCA, and OV than in normal tissues (Supplementary Figure S1C). The results revealed that *TOP1MT* is a carcinogenic molecule.

### The relationship between *TOP1MT* expression and tumor prognosis

**FIGURE 2:**
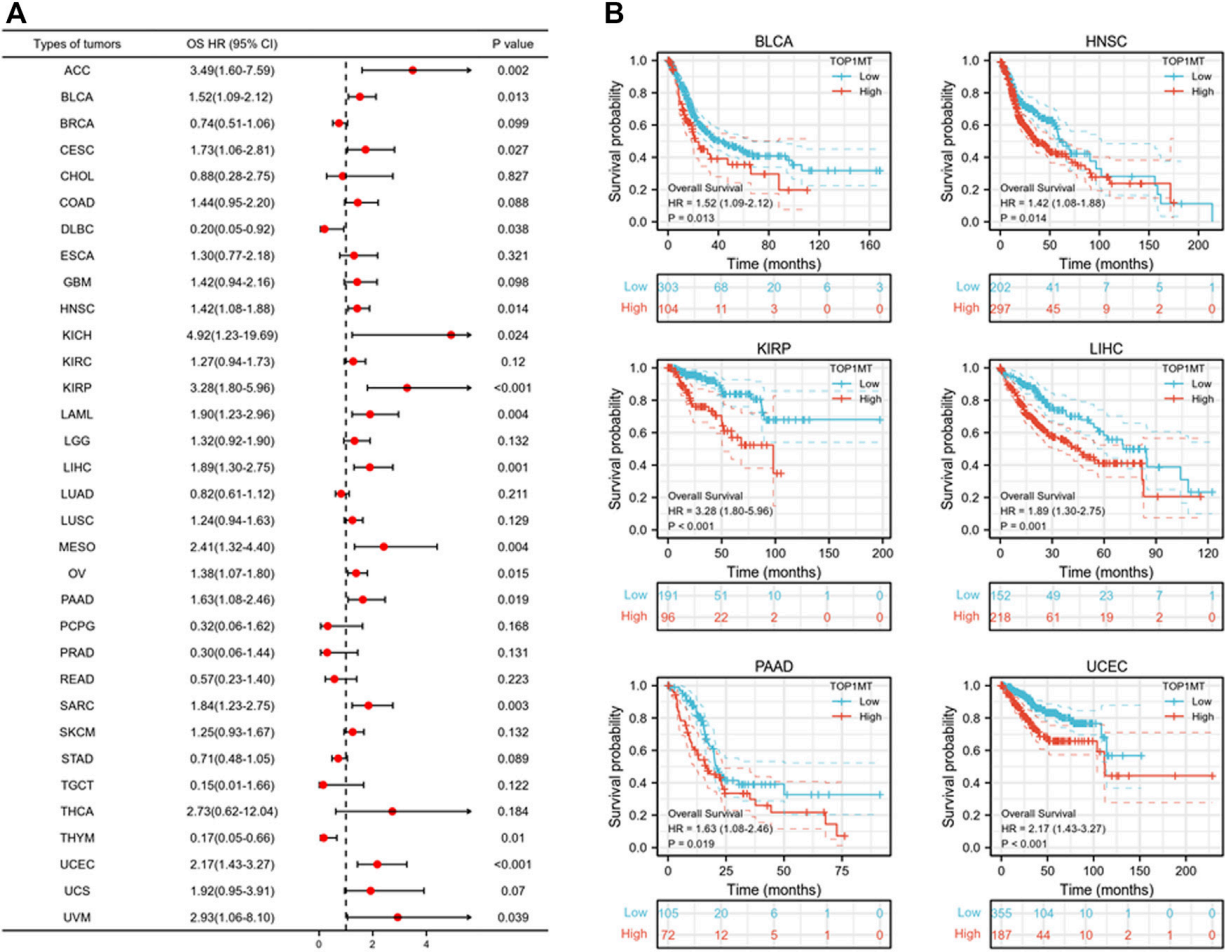
Relationship between *TOP1MT* expression and OS in cancer patients. **(A)** Forest map of *TOP1MT* risk ratios in 33 tumors; **(B)** Kaplan-Meier survival curves of OS in BLCA, HNSC, KIPP, PAAD, UCEC, and LIHC patients. ∗ *p* < 0.05, ∗∗ *p* < 0.01 and ∗∗ *p* < 0.001.

**FIGURE 3:**
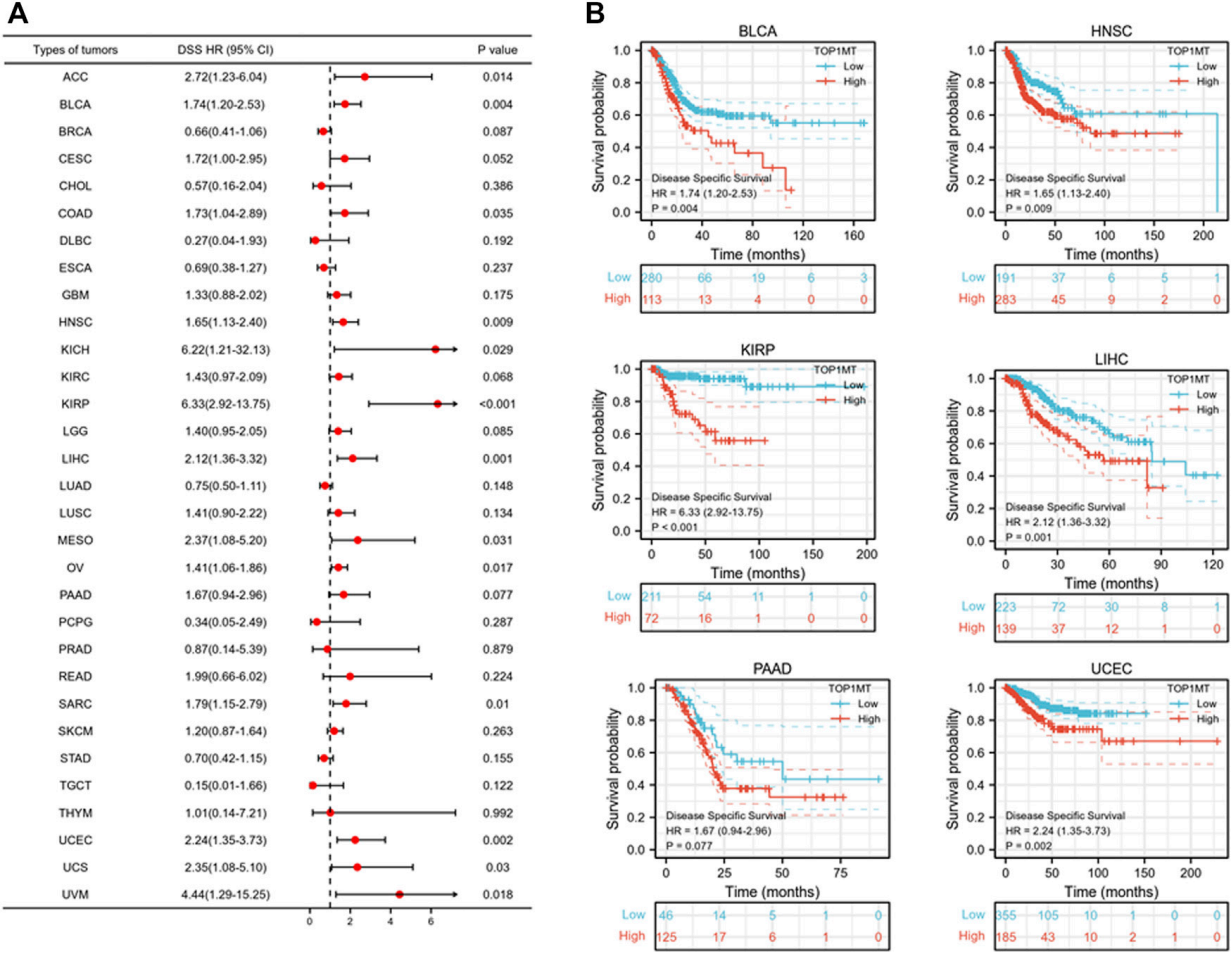
Relationship between *TOP1MT* expression and DSS in cancer patients. **(A)** Forest map of *TOP1MT* risk ratios in 33 tumors; **(B)** Kaplan-Meier survival curve of DSS in PATIENTS with BLCA, HNSC, KIPP, PAAD, UCEC, and LIHC. ∗ *p* < 0.05, ∗∗ *p* < 0.01 and ∗∗ *p* < 0.001.

**FIGURE 4:**
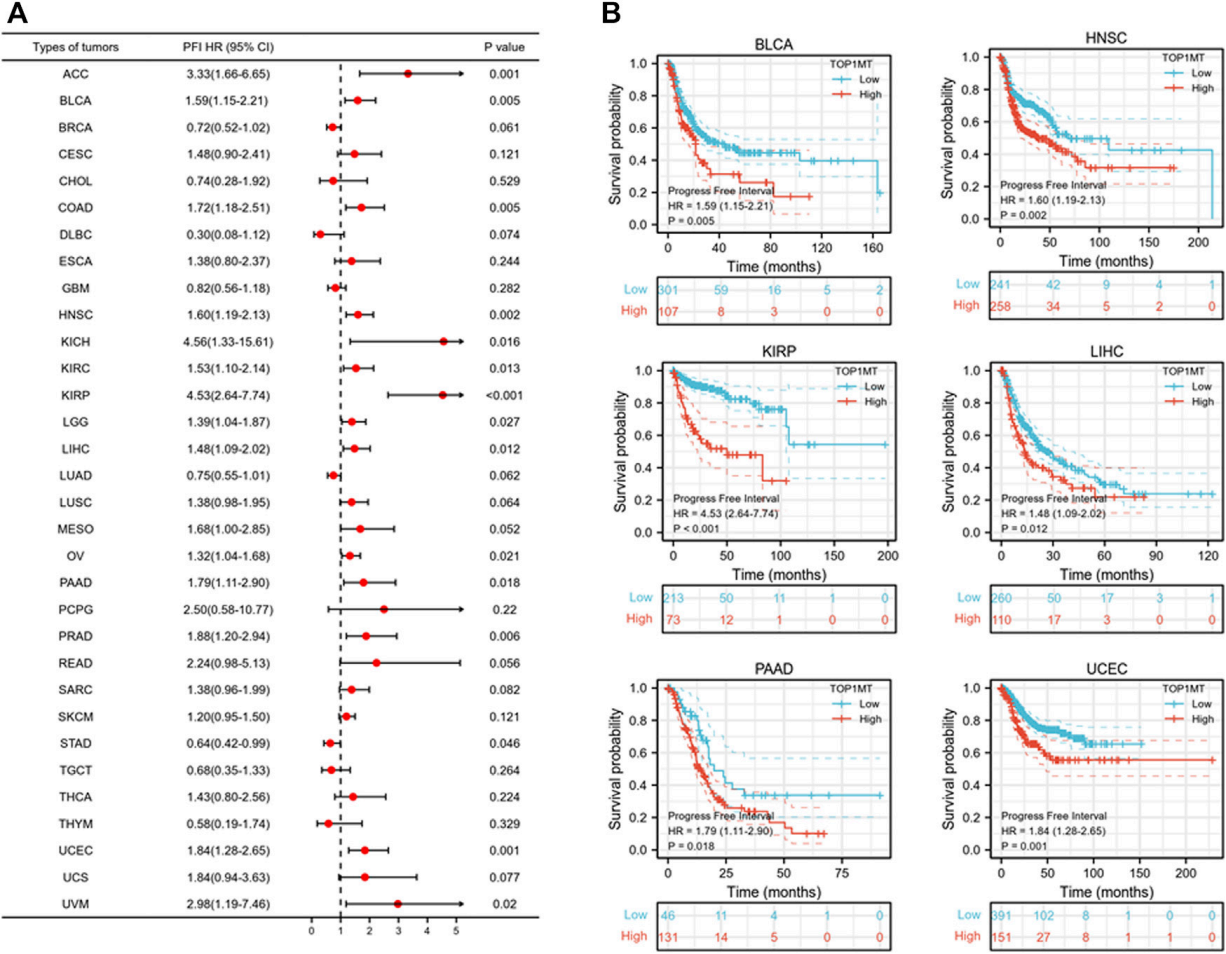
Relationship between *TOP1MT* expression and PFI in cancer patients. **(A)** Forest map of *TOP1MT* risk ratios in 33 tumors; **(B)** Kaplan-Meier survival curves of PFI in PATIENTS with BLCA, HNSC, KIPP, PAAD, UCEC, and LIHC. ∗ *p* < 0.05, ∗∗ *p* < 0.01 and ∗∗ *p* < 0.001.

Further, we investigated the effect of abnormal *TOPIMT* expression on prognosis. Survival indicators included OS, DSS, and PFI. A Cox regression analysis of 33 cancers revealed that *TOP1MT* expression significantly correlated with OS in 16, including ACC, BLCA, CESC, DLBC, HNSC, KICH, KIPP, LAML, LIHC, MESO, OV, PAAD, SARC, THYM, UCEC, and UVM (Figure 2A). The Kaplan-Meier survival curve revealed that upregulated *TOPIMT* expression in BLCA, HNSC, KIPP, PAAD, UCEC, and LIHC significantly correlated with poor overall survival (Figure 2B). Moreover, up-regulation of *TOPIMT* expression was significantly associated with low OS in ACC, CESC, DLBC, KICH, LAML, MESO, OV, SARC, THYM, and UVM cancer tissues; whereas high *TOP1MT* expression was significantly associated with high prognosis in DLBC and THYM cancer tissues (Supplementary Figure S2A). This work further investigated the relationship between *TOPIMT* expression and DSS in cancer patients. *TOPIMT* expression affected DSS in 13 cancers, including ACC, BLCA, COAD, HNSC, KICH, KIPP, LIHC, MESO, OV, SARC, UCUE, UCS, and UVM (Figure 3A). Kaplan-Meier analysis revealed that upregulated *TOPIMT* expression was associated with poor DSS in patients with BLCA, HNSC, KIPP, UCEC, and LIHC, but without any significant prognostic value for PAAD (Figure 3B). Also, ACC, COAD, KICH, MESO, OV, SARC, and UVM had poor prognostic values (Supplementary Figure S2B). Cox regression analysis of PFI revealed that upregulated *TOPIMT* expression was a risk factor for ACC, BLCA, COAD, HNSC, KICH, KIRC, KIPP, LGG, LIHC, OV, PAAD, PRAD, STAD, UCEC, and UVM (Figure 4A). Kaplan-Meier analysis demonstrated a relationship between upregulated *TOPIMT* expression and poor prognosis in six types of cancer, including BLCA, HNSC, KIPP, PAAD, UCEC, and LIHC (Figure 4B). Furthermore, upregulated *TOPIMT* expression contributed to adverse prognosis in ACC, COAD, KICH, KIRC, LGG, OV, PAAD, STAD, and UVM (Supplementary Figure S2C). Eventually, Kaplan-Meier Plotter database analysis revealed that high expression in BLCA, HNSC, KIPP, PAAD, UCEC, and LIHC tumor tissues confirmed poor prognosis (Supplementary Figure S3). We identified the six most significant cancers by combining the expression features of *TOP1MT* mRNA in pan-cancerous tissues and prognostic analysis of OS, DSS, and PFI. These cancers include BLCA, HNSC, KIRP, PAAD, UCEC, and LIHC. High *TOP1MT* expression in these six types of tumor tissues was significantly linked to poor prognosis.

**FIGURE 5:**
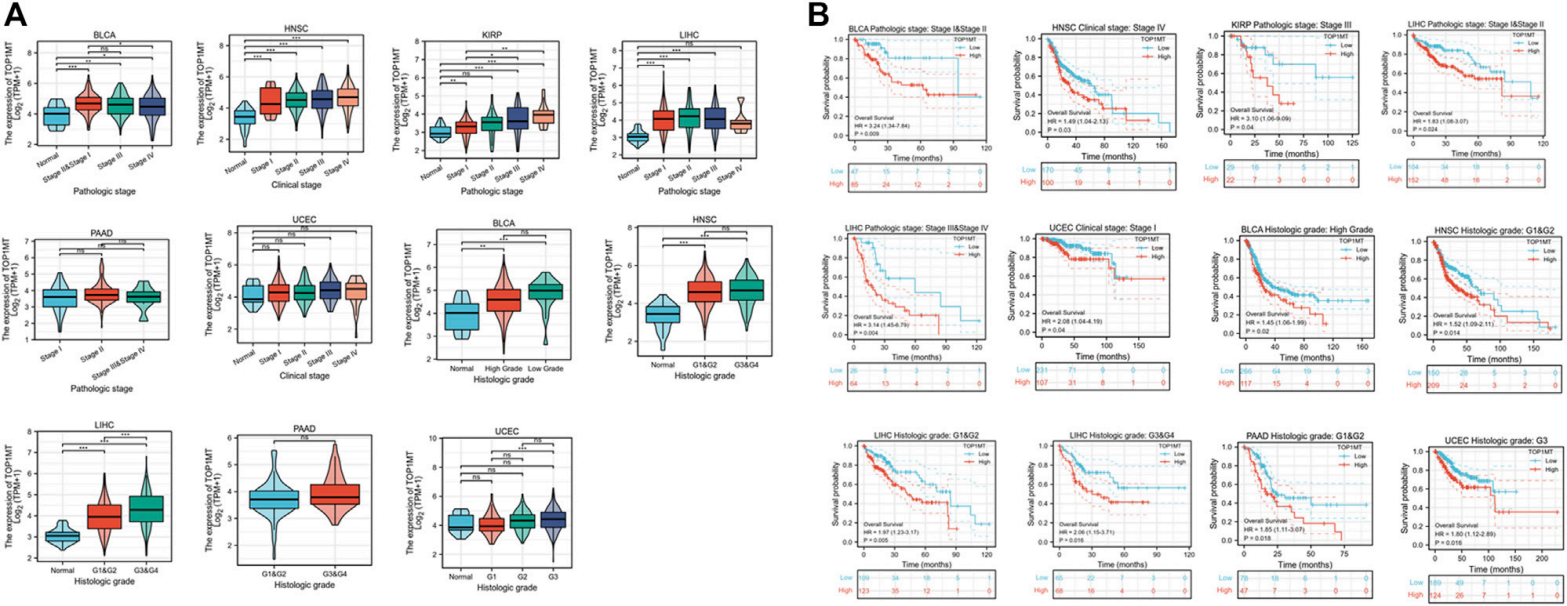
Correlation between *TOP1MT* expression, primary pathological stages, and prognosis. **(A)** Relationship between *TOP1MT* mRNA expression and different pathological stages in different cancer patients from TCGA; **(B)** Kaplan-Meier survival curves of *TOP1MT* mRNA expression in different pathological stages. Log_2_ (TPM+1) is used for the logarithmic scale. ∗ *p* < 0.05, ∗∗ *p* < 0.01 and ∗∗ *p* < 0.001.

Secondly, *TOPIMT* expression was examined in cancer patients with various pathological stages and grades to investigate the expressionist relationship with clinic pathological features. The TCGA database was used to evaluate *TOP1MT* expression in patients with stage I, II, III, and IV cancers, as well as grades G1, G2, G3, and G4. *TOP1MT* expression was significantly up-regulated in poor pathological features of BLCA, HNSC, KIPP, PAAD, UCEC, and LIHC (Figure 5A). The Kaplan Meier survival curve analysis revealed that poor pathological stages and grades exhibited a low overall survival (Figure 5B). Finally, the expression features of tumor size, lymph node status, and distant metastasis were investigated between *TOP1MT* and BLCA, HNSC, KIRP, PAAD, and LIHC using the TCGA database. The higher *TOP1MT* expression in BLCA, HNSC, KIRP, PAAD, and LIHC tumor tissues, the larger tumor diameter, the wider range of lymph node involvement, and the distant organ metastasis (Supplementary Figure S4A). Further, the relationship between *TOP1MT* expression and TNM stage prognosis was investigated using the Kaplan-Meier survival curve. The specific manifestations include: High expression of T1&T2 and N0 in BLCA patients indicates a poor prognosis. High expression in T3&T4, lymph node-positive, and M0 patients in HNSA suggested a poor prognosis. High expression in T1&T2 and T3&T4 in KIPP suggested a poor prognosis. High expression in T1&T2, T3&T4, N0, and M0 patients in LIHC suggests a poor prognosis. Patients with PAAD expressing high levels of T1&T2 and N1 had a poor prognosis (Supplementary Figure S4B).

### The relationship between *TOP1MT* expression, methylation level, and copy number changes for pan-cancer

**FIGURE 6:**
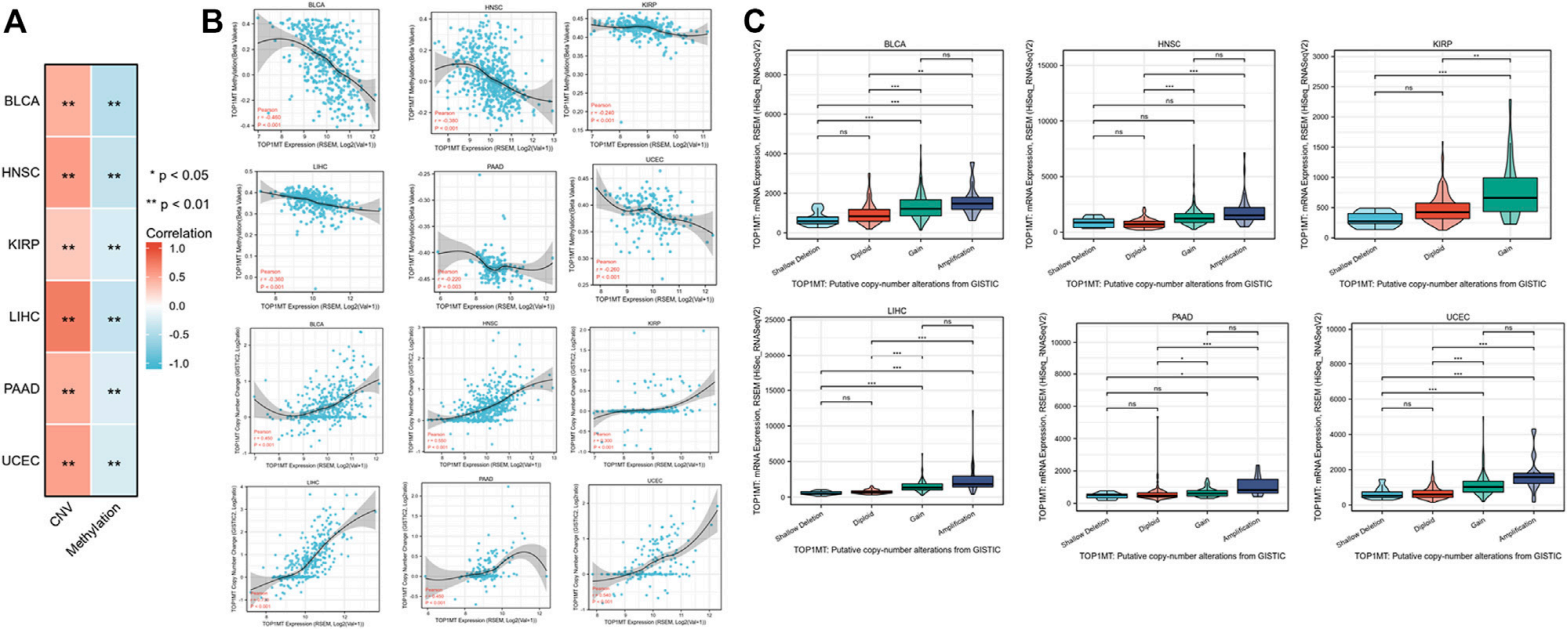
CNA and DNA methylation of *TOP1MT* in human cancer. **(A,B)** Linkedomics analysis correlation analysis between *TOP1MT* expression and *TOP1MT* methylation level and copy number variation data level; **(C)** cBioPortal analysis of *TOP1MT* CAN data in different cancer studies. ∗ *p* < 0.05, ∗∗ *p* < 0.01 and ∗∗ *p* < 0.001.

Linkedomics database was used to analyze the methylation level and copy number changes of *TOP1MT* in BLCA, HNSC, KIRP, PAAD, UCEC, and LIHC tumor tissues to understand the mechanism of the *TOP1MT* gene during tumorigenesis. Consequently, tumor tissues including BLCA, HNSC, KIPP, PAAD, UCEC, and LIHC significantly correlated with *TOPIMT* methylation and gene copy number levels (Figure 6A). *TOP1MT* expression positively correlated with CNA in BLCA, HNSC, KIRP, PAAD, UCEC, and LIHC tumors, whereas negatively correlated with methylation in BLCA, HNSC, KIRP, PAAD, UCEC, and LIHC tumors (Figure 6B). The relationship between *TOP1MT* genetic changes and mRNA expression on the cBioPortal website was explored using the GISTIC algorithm. As illustrated in Figure 6C, increased *TOP1MT* copies (gain and amplification) in tumor tissues significantly increased *TOP1MT* mRNA expression unlike copy neutral (diploid) and copy loss (shallow deletion). These results show that *TOP1MT* mRNA expression is affected by changes in its DNA copy number. Thus, abnormal *TOP1MT*mRNA expression in BLCA, HNSC, KIRP, PAAD, UCEC, and LIHC tumor tissues could be attributed to a high copy number and low methylation level.

### Relationship between *TOPIMT* expression and immune infiltration

**FIGURE 7:**
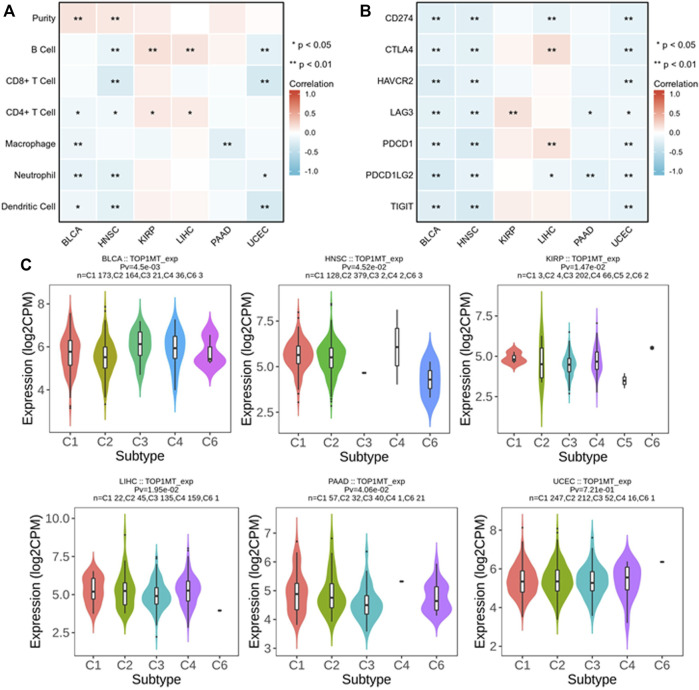
Correlation analysis between *TOP1MT* expression and tumor immune invasion. **(A)** *TOP1MT* expression significantly correlates with infiltration level of various immune cells in the TIMER database; **(B)** *TOP1MT* expression significantly correlates with various immune checkpoints in the TIMER database; **(C)** *TOP1MT* expression in different molecular subtypes of cancer by TISIDB. ∗ *p* < 0.05, ∗∗ *p* < 0.01 and ∗∗ *p* < 0.001.

We established the prognostic value of *TOPIMT* and investigated its relationship with tumor-infiltrating immune cells using the TIMER database. *TOPIMT* expression significantly correlated with the abundance of infiltrated immune cells (Figure 7A). The clustering heat map of the relationship between *TOP1MT* and immune cells demonstrated a positive correlation of *TOP1MT* with B cells and CD4+T lymphocytes in KIPP and LIHC cancers and with purity in BLCA and HNCC. *TOP1MT* negatively correlated with B cells and CD8+T lymphocytes in HNSC and UCUE, neutrophil cells and dendritic cells in BLCA, HNSC, and UCUE, CD4+T lymphocytes in BLCA and HNSC, and macrophages in BLCA and PAAD. Supplementary Figure S5 shows a specific correlation between each tumor and immune cells. Immune surveillance is widely recognized as important for cancer prognosis, and tumors can evade immune responses utilizing immune checkpoint genes. The correlation of *TOP1MT* with the expression of immune checkpoint genes was examined to investigate the relationship between *TOP1MT* expression and the degree of immune invasion in BLCA, HNSC, KIRP, and PAAD, UCEC, and LIHC. *TOP1MT* expression negatively correlated with common immune checkpoints in BLCA, HNSC, and UCUE (Figure 7B). Also, *TOP1MT* expression positively correlated with LAG3 in KIRP and negatively correlated with LAG3 and PDCD1LG2 in PAAD. *TOP1MT* positively correlated with CTLA4 and PDCD1 and negatively correlated with CD274 and PDCD1LG2 in LIHC. These findings suggest that high *TOP1MT* expression modulates immune invasion of tumor tissues, especially BLCA, HNSC, and UCUE tumor tissues.

The expression of the *TOP1MT* gene in different immune subtypes was analyzed using the TISIDB database. Significant differences in *TOPIMT* expression characteristics were noted in the C1 (wound healing), C2 (IFN-γ dominant), C3 (inflammation), C4 (lymphocytopenia), C5 (immune silencing), and C6 (TGF-β dominant) subtypes in the six cancer tissues (Figure 7C). *TOP1MT* was highly expressed in C1 of BLCA, PAAD, UCEC, C2 of HNSC, C3 of KIRP, and C4 of LIHC. Differential *TOP1MT* expression in different immune subtypes predicts that *it* regulates cancer prognosis (Thorsson et al., 2019).

### Functional analysis of *TOPIMT*

**FIGURE 8:**
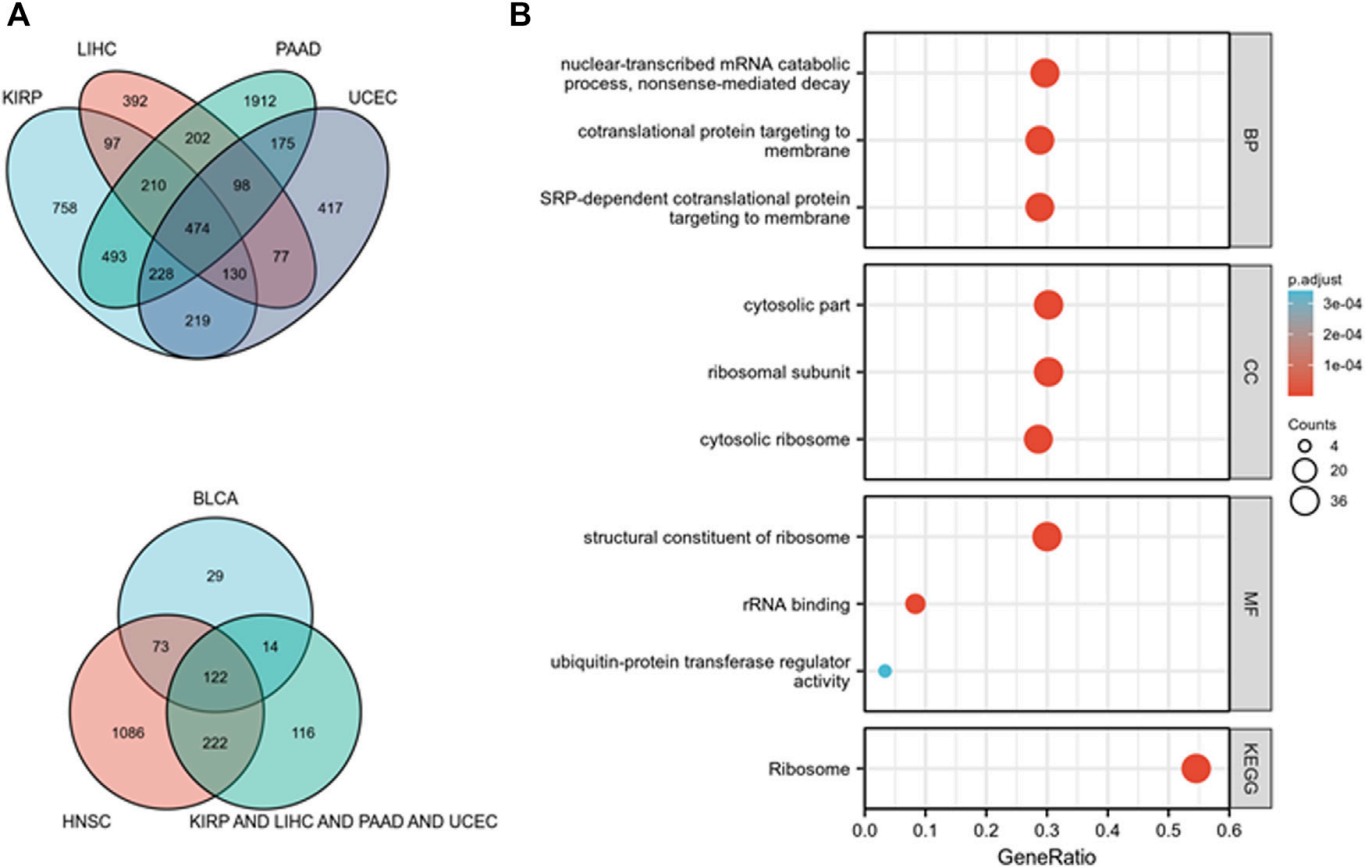
*TOP1MT* functional analysis. **(A)** Co-expressed genes in BLCA, HNSC, KIPP, PAAD, UCEC, and LIHC tumors; **(B)** KEGG and GO pathway analysis of co-expressed genes in BLCA, HNSC, KIPP, PAAD, UCEC, and LIHC tumor tissues.

KEGG and GO pathway analyses were performed on multiple cancer types to reveal the potential function of *TOPIMT*. First, we mapped genes expressed in pan-cancer tissues using the TCGA database and identified 122 co-expressed genes in six cancer tissues (Figure 8A). Co-expressed genes were used to examine KEGG and GO pathways. As shown in Figure 8B, *TOP1MT* modulates the ribosomal pathway of genetic information. As per the GO analysis, *TOP1MT* affected ribosomal associated BP, CC, and MF in most cancers, including BP (nuclear transcriptional mRNA catabolism process, membrane co-transfer protein, membrane-targeted SRP-dependent co-translation protein, and other biological processes); CC (cytoplasmic part, ribosomal subunit, cytoplasmic ribosome, and other cellular components); MF (rRNA linkage, structural composition of ribosomes, regulatory activity of ubiquitin-protein transferase and other molecular functions).

## Discussion

Mitochondrial topoisomerase I (*TOP1MT*) is a type IB topoisomerase that exists in vertebrates and specifically targets mitochondria (Zhang et al., 2001; Pommier et al., 2016; Baechler et al., 2019). *TOP1MT* relaxes the mitochondrial DNA (mtDNA) superhelix by introducing instantaneous cutting complexes, revolving broken DNA strands around intact strands (Zhang et al., 2001; Dalla Rosa et al., 2014). In addition to regulating mtDNA, *TOP1MT* directly affects mitochondrial translation through protein interactions with small mitochondrial glycosomal subunits. *TOP1MT* inhibitors may be an alternative approach for targeting mitochondrial DNA due to their role in mitochondrial protein synthesis and their up-regulation in several tumors (Khiati et al., 2014). Nonetheless, we did not conduct a pan-cancer analysis on the function of *TOP1MT* in different cancers.

This work examined *TOP1MT* expression in pan-cancer datasets. In an analysis of 33 cancer datasets obtained from the TCGA, *TOP1MT* expression was significantly higher in BLCA, BRCA, CHOL, COAD, DLBC, ESCA, GBM, HNSC, KIRC, KIRP, LGG, LIHC, LUAD, LUSC, PAAD, PCPG, PRAD, READ, SKCM, TAD, THYM, UCEC, UC than in normal tissues. No significant difference was noted between *TOP1MT* and normal tissue in tumor samples from ACC, CESC, LAML, and TGCT. Low *TOP1MT* expression was discovered in tumor samples from KICH, THCA, and OV than that in normal tissues. This means that *TOP1MT* could play different roles in different cancer types. Gene mutation is crucial in human cancer development. *TOP1MT* expression positively correlates with CNV. Moreover, *TOP1MT* upregulation was linked to the poor OS, DSS, and PFI in BLCA, HNSC, KIPP, PAAD, UCEC, as well as LIHC. DSS exhibited no prognostic value in PAAD cancer patients with upregulated *TOPIMT* expression. Additional analysis revealed that high *TOP1MT* expression was linked to poor overall survival in patients with poor clinic pathologic typing. These findings reveal the *TOP1MT* role in promoting tumor progression and that high *TOP1MT* expression could impair survival in cancer patients.

The tumor microenvironment (TME) encompasses tumor cells, immune cells, and stromal cells; it is affected by various factors including cytokines, reactive oxygen species, extracellular Matrix, metabolites, and inflammation (Chen et al., 2015; Balta et al., 2021). TME is fundamental in tumor inhibition or progression (Roma-Rodrigues et al., 2019). Humans have innate and adaptive immunity that combat various diseases, including cancer (Schreiber et al., 2011; Vesely et al., 2011; Seager et al., 2017). Tumor cells have evolved immune-evasion mechanisms among them loss of antigenicity, immunogenicity, and generation of immunosuppressive TME (Khong and Restifo, 2002; Blank et al., 2005; Thomas and Massague, 2005; Drake et al., 2006). Consequently, immunotherapy continues to face significant hurdles in cancer treatment (Murciano-Goroff et al., 2020; Zhang and Zhang, 2020)^.^ A total of seven common immune cells and immune checkpoints were selected to analyze the relationship between *TOP1MT* expression immune cells, and immune checkpoints. We found that *TOP1MT* expression is linked to immune invasion and checkpoint markers in BlCA, HNSC, KIPP, PAAD, UCEC, and LIHC cancers. *TOP1MT* is associated with tumor purity, B cells, CD8^+^ T cells, CD4^+^ T cells, macrophages, neutrophils, and dendritic cells in various cancers. Previous studies have shown that tumors with higher levels of Programmed cell death-Ligand 1(PD-L1) are more malignant and less survivable; besides, suppressing the PD-1/PD-L1 pathway promotes tumor cell survival (Nakanishi et al., 2007; Okudaira et al., 2009). Pai and colleagues proposed an anti-CTLA4 blocking technique that simultaneously reduces tumor invasion, maintains the anti-tumor effect, and minimizes toxicity (Pai et al., 2019). As a consequence, the expression of the checkpoint genes programmed cell death 1(PD-1), PD-L1, and cytotoxic T-lymphocyte-associated protein 4 (CTL-4) is a predictive biomarker of immunosuppressive response. Notably, *TOP1MT* and checkpoint gene expression were significantly correlated. *TOP1MT* expression negatively correlated with most immunosuppressive sites. These results indicate a potential relationship between *TOP1MT* and immunologic invasion in cancer patients. Collectively, our research sheds light on the application of *TOP1MT* as a potential prognostic biomarker for several cancers in the context of immuno-oncology, as well as a theoretical reference for new targets.

DNA methylation is an epigenetic mechanism necessary for gene transcription (Feinberg and Vogelstein, 1983; Hsieh and Gage, 2004). Of note, 5-methylcytosine is synthesized by attaching methyl groups to cytosine residues in cytosine-guanine (CG) (Patil et al., 2014; Mahmoud and Ali, 2019). Since most of the CpG sites in the human genome are methylated, low methylation of CpG sites in these regions induces genomic instability and loss of genomic imprinting. This ultimately results in tumor cell development (Kulis et al., 2013). Also, hypermethylation in the same promoter region silences or inactivates the tumor suppressor gene in cancer cells (Bakshi et al., 2018). Studies on *TOP1MT* methylation in cancer are limited. Our findings suggest a negative correlation between *TOP1MT* expression and DNA methylation, indicating *TOP1MT* expression in BlCA, HNSC, KIPP, PAAD, UCEC, and LIHC with low methylation. Moreover, the findings of this work act as a reference for further research on *TOP1MT* methylation-related roles, and expound on the *TOP1MT* mechanism in tumorigenesis and development.

*TOP1MT* ribosome functions are linked to the biological function of *TOP1MT* in patients with liver cancer (Baechler et al., 2019). Evidence shows that the biogenesis of mitochondrial ribosomes depends on the coordinated synthesis of 80 mitochondrial ribosomal proteins encoded in nuclear DNA, which must be translated into mitochondria via the cytoplasmic ribosomes (Bogenhagen et al., 2014). Therefore, the formation of mitochondrial glycosomes is critical for subsequent mitochondrial function. Biological function analysis revealed that the function of *TOP1MT* is primarily related to ribosome-related activities. Notably, *TOP1MT* significantly modulates mitochondrial function.

Although we used data from multiple databases to analyze the significance of *TOP1MT*, this work has compelling limitations. First, we searched the database for phenotypic features of *TOP1MT* expression in various cancers, yet no additional evidence was discovered in the cell, animal, or clinical samples. Secondly, we verified that *TOP1MT* expression is associated with immunologic invasion. Nevertheless, we did not find any in-depth study on TOP1MT and immune infiltration in relevant articles, which lacked certain basis -. Thus, the specific approach by which *TOP1MT* participates in immune infiltration is required. Furthermore, while we found that the biological functions of *TOP1MT* of 122 co-expressed genes in six cancer tissues were related to ribosomal functions, limitations were noted. Therefore, additional studies on other promising biological roles of *TOP1MT* are necessary.

In conclusion, this is the first comprehensive examination of *TOP1MT* in pan-cancer. The results demonstrate a heterologous *TOP1MT* expression in various cancers; besides, upregulated *TOP1MT* expression is associated with poor prognosis. Moreover, *TOP1MT* expression significantly correlates with immune invasion. Thus, *TOP1MT* is a potential biomarker for mitochondrial anticancer therapy and cancer immunotherapy.

## Data Availability

The original contributions presented in the study are included in the article/Supplementary Material, further inquiries can be directed to the corresponding authors.
