# Supplementary material for: A comprehensive pan-cancer analysis of the expression characteristics, prognostic value, and immune characteristics of TOP1MT
Source: Front Genet. 2022 Aug 10;13:920897. doi: 10.3389/fgene.2022.920897 (PMC9399363; doi:10.3389/fgene.2022.920897)
Supplement: Supplementary file 1 [file Table1.docx]

Supplement Table 1: Sample size of 33 different tumor types

| Group1 | Group2 | Number |
| --- | --- | --- |
| ACC | Normal | 128 |
| ACC | Tumor | 77 |
| BLCA | Normal | 28 |
| BLCA | Tumor | 407 |
| BRCA | Normal | 292 |
| BRCA | Tumor | 1099 |
| CESC | Normal | 13 |
| CESC | Tumor | 306 |
| CHOL | Normal | 9 |
| CHOL | Tumor | 36 |
| COAD | Normal | 349 |
| COAD | Tumor | 290 |
| DLBC | Normal | 444 |
| DLBC | Tumor | 47 |
| ESCA | Normal | 666 |
| ESCA | Tumor | 182 |
| GBM | Normal | 1157 |
| GBM | Tumor | 166 |
| HNSC | Normal | 44 |
| HNSC | Tumor | 520 |
| KICH | Normal | 53 |
| KICH | Tumor | 66 |
| KIRC | Normal | 100 |
| KIRC | Tumor | 531 |
| KIRP | Normal | 60 |
| KIRP | Tumor | 289 |
| LAML | Normal | 70 |
| LAML | Tumor | 173 |
| LGG | Normal | 1152 |
| LGG | Tumor | 523 |
| LIHC | Normal | 160 |
| LIHC | Tumor | 371 |
| LUAD | Normal | 347 |
| LUAD | Tumor | 515 |
| LUSC | Normal | 338 |
| LUSC | Tumor | 498 |
| MESO | Tumor | 87 |
| OV | Normal | 88 |
| OV | Tumor | 427 |
| PAAD | Normal | 171 |
| PAAD | Tumor | 179 |
| PCPG | Normal | 3 |
| PCPG | Tumor | 182 |
| PRAD | Normal | 152 |
| PRAD | Tumor | 496 |
| READ | Normal | 318 |
| READ | Tumor | 93 |
| SARC | Normal | 2 |
| SARC | Tumor | 262 |
| SKCM | Normal | 813 |
| SKCM | Tumor | 469 |
| STAD | Normal | 210 |
| STAD | Tumor | 414 |
| TGCT | Normal | 165 |
| TGCT | Tumor | 154 |
| THCA | Normal | 338 |
| THCA | Tumor | 512 |
| THYM | Normal | 446 |
| THYM | Tumor | 119 |
| UCEC | Normal | 101 |
| UCEC | Tumor | 181 |
| UCS | Normal | 78 |
| UCS | Tumor | 57 |
| UVM | Tumor | 79 |
