# Supplementary material for: A comprehensive pan-cancer analysis of the expression characteristics, prognostic value, and immune characteristics of TOP1MT
Source: Front Genet. 2022 Aug 10;13:920897. doi: 10.3389/fgene.2022.920897 (PMC9399363; doi:10.3389/fgene.2022.920897)

Supplement Figure 1:

TOP1MT mRNA expression in different cancer patients from TCGA. ∗ P < 0.05, ∗∗ P < 0.01 and ∗∗ P < 0.001


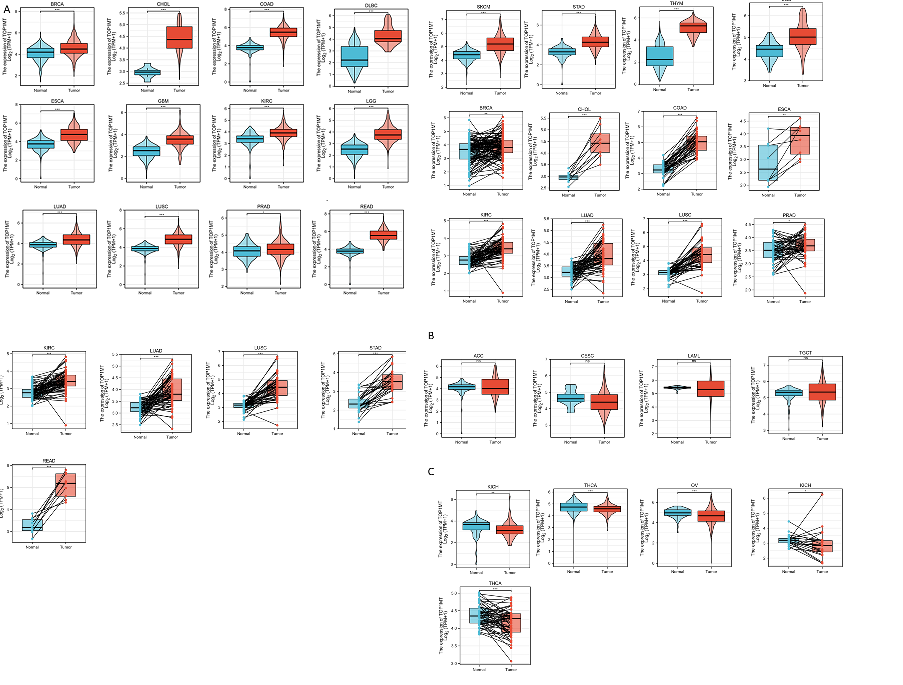


Supplement Figure 2: Association between TOP1MT expression and Kaplan-Meier survival curve in cancer patients.


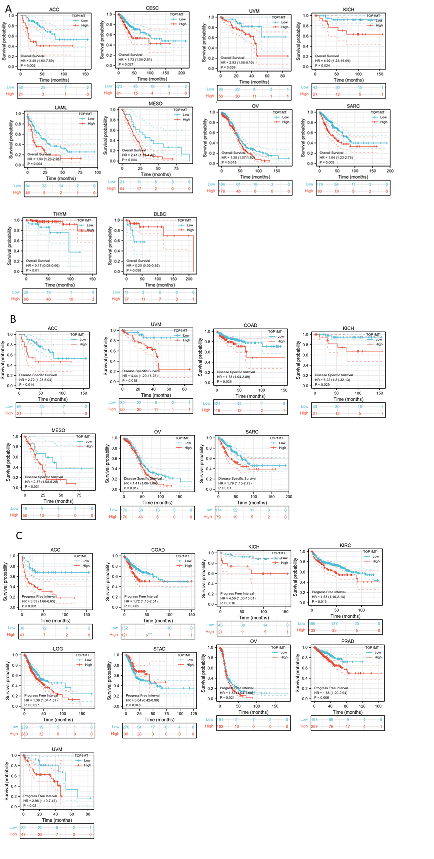


Supplement Figure 3: Analysis of TOP1MT mRNA expression in patients with BLCA, HNSC, KIPP, PAAD, UCEC, and LIHC using the Kaplan-Meier Plotter database.

∗ P < 0.05, ∗∗ P < 0.01 and ∗∗ P < 0.001


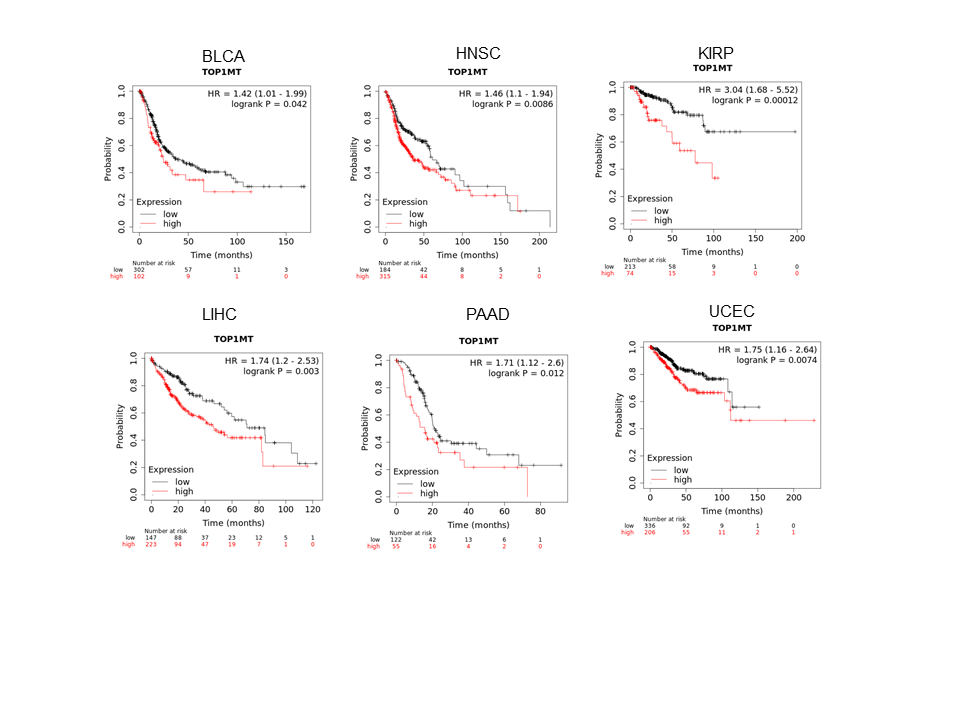


Supplement Figure 4: Analysis of TOP1M expression and different pathological features and prognosis of BLCA, HNSC, KIPP, PAAD, and LIHC cancers.

∗ P < 0.05, ∗∗ P < 0.01 and ∗∗ P < 0.001


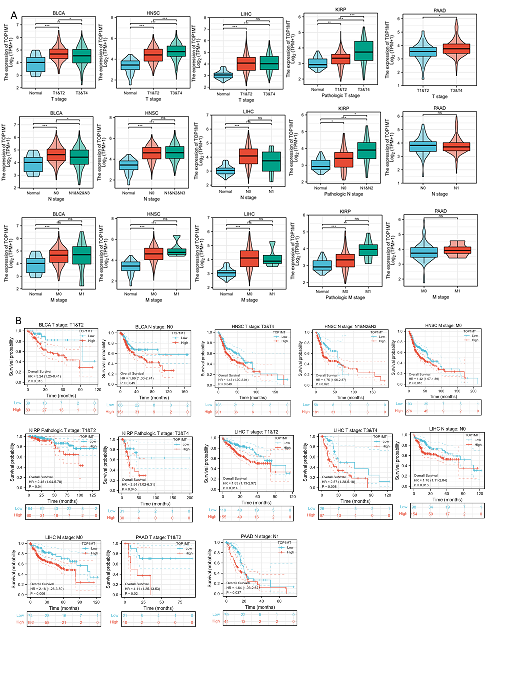


Supplement Figure 5: Correlation analysis of TOP1MT expression with immune invasion in patients with BLCA, HNSC, KIPP, PAAD, UCEC, and LIHC cancer


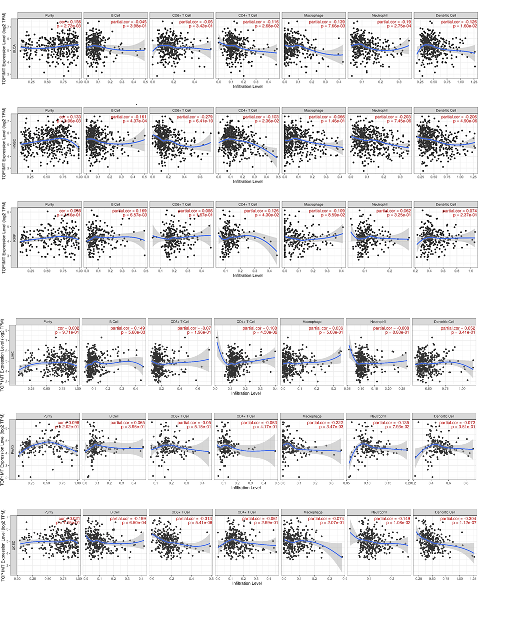

Supplement: Supplementary file 2 [file DataSheet1.docx]
